# Association of Subclinical Hypothyroidism with Present and Absent Anti-Thyroid Antibodies with PCOS Phenotypes and Metabolic Profile

**DOI:** 10.3390/jcm11061547

**Published:** 2022-03-11

**Authors:** Iwona Magdalena Gawron, Rafał Baran, Kamil Derbisz, Robert Jach

**Affiliations:** 1 Department of Obstetrics and Gynecology, Jagiellonian University Medical College, 31-008 Kraków, Poland; jach@cm-uj.krakow.pl; 2 Clinical Department of Gynecological Endocrinology and Gynecology, University Hospital in Krakow, 31-501 Kraków, Poland; baran.rafal@me.com (R.B.); kamder92@gmail.com (K.D.)

**Keywords:** subclinical hypothyroidism, polycystic ovary syndrome, insulin resistance

## Abstract

Background: Polycystic ovary syndrome (PCOS) and subclinical hypothyroidism (SCH) often coexist, but implications of the co-occurrence of two disorders have not yet been established. The objective is to conclude whether SCH with present or absent anti-thyroid antibodies (ATA) impacts on the PCOS phenotype and alters biochemical or clinical parameters. Methods: A retrospective cohort study was conducted in a tertiary reference center. Clinical and biochemical parameters of women with PCOS were analyzed. Results: A total of 367 women with PCOS were included in the study, 114 (31.1%) of whom were diagnosed with SCH and 16 (4.4%) with autoimmune thyroiditis (AIT). Among all parameters studied, the strongest relationship with SCH was confirmed for insulin resistance and dyslipidemia. SCH was an independent risk factor for insulin resistance. In SCH the additional presence of ATA did not exacerbate the metabolic disorders. There was no significant association of any PCOS phenotype with SCH, nor with the presence of circulating ATA. There was no significant difference in hormonal parameters and mFerriman–Gallwey scale score between women with PCOS with and without SCH. Conclusions: SCH alters metabolic, but not hormonal, parameters in PCOS. The diagnosis of SCH does not exclude the diagnosis of PCOS. The potential effect of positive ATA was insignificant.

## 1. Introduction

Polycystic ovary syndrome (PCOS) is one of the most common endocrinopathies and affects 3 to 15% of women of reproductive age, depending on the studied population and the diagnostic criteria used [1,2]. The most widely accepted diagnostic criteria of PCOS, i.e., the Rotterdam criteria [3], require the presence of two of the three following clinical symptoms: (i) hyperandrogenism/ hyperandrogenemia, (ii) ovulation disorders, i.e., oligoovulation or anovulation, or (iii) polycystic ovaries on ultrasound examination, while excluding other conditions that may cause hyperandrogenism or ovulation dysfunction. The exact etiology of PCOS has not yet been fully understood, and the results of scientific studies suggest the involvement of genetic, metabolic, immunological and environmental factors, likely already present in utero [4,5,6,7,8,9]. The syndrome may also be accompanied by other endocrine and metabolic disorders, contributing to an increased risk of endometrial cancer, obesity, diabetes, hypertension, dyslipidemia, and cardiovascular diseases [10,11]. Studies conducted in recent years also suggest a higher incidence of thyroid diseases, including subclinical hypothyroidism (SCH) [12,13] and autoimmune thyroiditis (AIT) [14] in women with PCOS, pointing out possible common etiology [15,16]. AIT, called Hashimoto thyroiditis (HT), affecting 5–20% of women of childbearing age, is one of the most common causes of hypothyroidism in areas with sufficient iodine content. The coexistence of AIT and PCOS concerns about 18–40% of all PCOS cases, depending on the diagnostic criteria of PCOS and ethnicity [17]. The similarity of clinical symptoms and complications between PCOS and thyroid dysfunctions, i.e., irregular menstrual cycles, subfertility, insulin resistance, obesity and dyslipidemia, is commonly recognized. Some studies indicate a relationship between thyroid autoimmunity and pregnancy outcomes such as miscarriage and preterm labor [18,19], as well as the outcomes of assisted reproductive techniques [20,21]. The coexistence of AIT with PCOS may be associated with an even greater risk of metabolic syndrome and infertility [22,23]. Complications pertain not only to overt hypothyroidism, but also SCH [24,25] and the presence of circulating anti-thyroid peroxidase (anti-TPO) and anti-thyroglobulin (anti-TG) antibodies [26]. However, not all research results are consistent in this respect. Some studies have not shown a higher prevalence of AIT in women with PCOS [27]. Similarly, not all research results confirmed the influence of thyroid disorders on the hormonal or metabolic profile of women with PCOS [27,28,29]. Reaching consensus on the correlation of AIT and PCOS, screening tests for the coexistence of these disorders and the timing of the initiation of possible treatment is necessary for the effective prevention of metabolic complications and infertility in women of reproductive age. The aim of this study was to estimate the prevalence of SCH and circulating anti-thyroid antibodies (ATA) in euthyroid women with PCOS. The specific objectives included: (i) comparison of selected metabolic, hormonal and clinical parameters in women with PCOS with and without thyroid dysfunction, and (ii) determination of PCOS phenotypes in women with and without thyroid dysfunction

## 2. Materials and Methods

A retrospective study was conducted based on the archival data of the Department of Gynecological Endocrinology between 1 January 2018 and 30 June 2021. The study was approved by the Bioethics Committee at the Regional Chamber of Physicians (positive opinion no. 215/KBL/OIL/2021). The study group consisted of women undergoing diagnostics due to menstrual disorders and/or infertility. The study included women diagnosed with E28 and E89 according to ICD-10 who met the Rotterdam ESHRE/ASRM-Sponsored PCOS criteria [3]. The inclusion criteria were (i) aged between 16-45 years, (ii) euthyreosis, and (iii) no previous diagnosis or treatment of hormonal disorders, while the exclusion criteria were (i) history of thyroidectomy, (ii) diagnosed and treated thyroid dysfunction, and (iii) diagnosed and treated states of insulin resistance or diabetes. Using archival medical records, the study population was characterized in terms of demographic data and thyroid function indicators, i.e., the concentration of thyroid-stimulating hormone (TSH) and ATA, including anti-TPO and anti-TG, and subsequently divided into subgroups: (i) TSH ≤ 2.5 [30] and anti-TPO, anti-TG within the reference range, (ii) TSH ≤ 2.5 and anti-TPO or/and anti-TG above reference range, (iii) TSH > 2.5 and anti-TPO, anti-TG within reference range, and (iv) TSH > 2.5 and anti-TPO and/or anti-TG above reference range. Then, the following parameters were analyzed for comparative analysis in the above-mentioned subgroups: (i) clinical parameters: menstrual cycle length, endometrial thickness, ovarian morphology, modified Ferriman–Gallwey scale (mFG) score [31]; (ii) hormonal parameters: free thyroxine (fT4), free triiodothyronine (fT3) luteinizing hormone (LH)/follicle-stimulating hormone (FSH) ratio; estradiol, free androgen index (FAI), testosterone, sex hormone-binding globulin (SHBG), prolactin, vitamin D; (iii) metabolic parameters: homeostasis model assessment for insulin resistance (HOMA-IR), 2 h 75 g oral glucose tolerance test results [32] (75 OGTT; glucose at 0, 60, 120 min, insulin at 0, 60, 120 min), triglycerides (TG), total cholesterol, low-density lipoprotein cholesterol (LDL), high-density lipoprotein cholesterol (HDL). Further, 21–35-day cycles were considered to be regular menstrual cycles. Vein blood samples were drawn in the early follicular phase (2nd–6th day of the cycle) or randomly in non-ovulatory cycles after an overnight fast of 8 h. Subsequent blood samples for 75 OGTT were taken at 60 and 120 min of rest after oral ingestion of the 75 g glucose solution. The diagnosis of insulin resistance (IR) was based on HOMA-IR > 2.5, fasting insulin level ≥ 13 µU/mL [33] and insulin at 120 min OGTT ≥ 100 µU/mL. The calculation formula for the homeostasis model assessment for insulin resistance (HOMA-IR) was the following: Score = (Fasting insulin [uIU/mL]) × (Fasting glucose [mg/dL])/405 [34]. AIT was diagnosed on the basis of TSH > 2.5 and the presence of anti-TPO and/or anti-TG antibodies. Insulin, glucose, TSH, fT3, fT4, anti-TG, anti-TPO, FSH, LH, testosterone, estradiol, SHBG, prolactin, vitamin D, C-reactive protein (CRP), cancer antigen-125 (Ca-125) measurements were performed using the electrochemiluminescence method by means of the Cobas PRO/e801 and Cobas 8000 analyzers (Roche Diagnostics, Basel, Switzerland). Cholesterol, HDL, LDL and TG measurements were performed using the enzymatic method by means of the Cobas PRO analyzer (Roche Diagnostics, Basel, Switzerland).

### 2.1. PCOS Diagnostics

The women included in the study underwent routine gynecological and hormonal diagnostics, consisting of a routine gynecological examination, ultrasound examination of the reproductive organs and blood sampling for selected laboratory tests used in the diagnostics of ovulation disorders. The gynecological examination was carried out by a specialist in obstetrics and gynecology or by a trainee physician supervised by a specialist obstetrician–gynecologist. The ultrasound examination was performed using a Samsung WS80A machine (Samsung Electronics, Suwon, Korea) with the use of a vaginal and/or transabdominal transducer. The presence of at least 12 circumferentially located follicles 2–9 mm in diameter or an ovarian volume > 10 mL was considered as a result indicative of a polycystic morphology of the ovaries. The mFG scale [31] was used to assess the subjective severity of hirsutism and the cut-off point indicative of clinical hyperandrogenism was defined as a score of at least 8 points. The Rotterdam ESHRE/ASRM-Sponsored [3] criteria were used to confirm PCOS. Taking into account various clinical features and laboratory parameters, 4 basic phenotypes of the PCOS syndrome were distinguished: (i) A—hyperandrogenism (increased testosterone serum level or FAI > 5 or mFG scale score ≥ 8) + irregular menstrual cycles + polycystic morphology of the ovaries; (ii) B—hyperandrogenism (increased testosterone serum level or FAI > 5 or mFG scale score ≥ 8) + irregular menstrual cycles; (iii) C—hyperandrogenism (increased testosterone serum level or FAI > 5 or mFG scale score ≥ 8) + polycystic morphology of the ovaries; (iv) D—irregular menstrual cycles + polycystic morphology of the ovaries. Written and oral consent was obtained for the above-mentioned tests. 

### 2.2. Statistical Analysis

Descriptive statistics were used to characterize the population. Categorical (qualitative) variables were summarized as number of cases (N, *n*), frequency (*n*/N), and percentage (%). Continuous (quantitative) variables were presented using means and standard deviations in the case of normal distribution and medians, upper and lower quartiles in other cases. The maximum and minimum values of the variables were also given. The normality of the variable distribution was checked with the Kolmogorov–Smirnov test. In order to compare the selected groups in the context of the assessed variables, the Mann–Whitney U test was used as a non-parametric test, and the Student’s t-test was used in the case of normal distribution. The testing of nominal and ordinal variables was performed using the chi square test. In the next step, the logistic regression was used to estimate the associations of variables with adverse metabolic and hormonal effects. The results were presented as odds ratios with 95% confidence intervals. Multivariable analysis was performed using a backward stepwise regression model to determine contributions of variables to significant metabolic outcomes. Testing of the significance of the regression coefficient was performed using the Wald statistic. An ROC curve was performed to assess the cut-off points for significant quantitative variables. A *p*-value < 0.05 was considered statistically significant. Statistical analysis was performed using StatSoft STATISTICA v.13.3 software.

## 3. Results

During the study period, the diagnosis of PCOS was confirmed in 429 women, and 367 (85.5%) of them met the inclusion criteria. SCH was confirmed in 114 women (*n* = 114/367; 31.1%), the presence of circulating ATA in 44 women (*n* = 44/367; 12.0%) and AIT in 16 (*n* = 16/367; 4.4%). The characteristics of the study population in relation to selected clinical, hormonal and metabolic parameters, as well as qualitative variables, are presented in Table 1 and Table 2. The characteristics of the subpopulations in terms of TSH values and ATA status in relation to selected quantitative and qualitative parameters are presented in Supplementary Tables S1 and S2. The subpopulation with TSH > 2.5 and positive ATA, anti-TPO and/or anti-TG included 16 women (*n* = 16/367; 4.4%). A statistically significantly lower value of endometrial thickness, higher LH/FSH ratio, higher fasting insulin concentration and higher HOMA-IR values compared to the rest of the study population are noteworthy. However, no association with any of the qualitative features was found. The subpopulation with TSH > 2.5 and negative ATA included 98 women (*n* = 98/367; 26.7%). In this group, statistically significantly higher glucose concentrations at 60 and 120 min of the 75OGTT, higher fasting insulin levels at 60 and 120 min of the 75OGTT, higher HOMA-IR, higher FAI, higher total cholesterol, higher total cholesterol/HDL ratio, higher LDL and LDL/HDL ratio, and higher triglyceride levels were found. A statistically significant correlation of the occurrence of insulin resistance was also confirmed. When analyzing the study population based only on TSH > 2.5 regardless of ATA status (*n* = 114/367; 31.1%), similar results were obtained, i.e., statistically significantly higher glucose concentration in the 120th minute of the 75OGTT, significantly higher insulin concentration at all 75 OGTT points, higher values of HOMA-IR and FAI indices and significantly higher values of total cholesterol, LDL and triglycerides and higher values of total cholesterol/HDL and LDL/HDL ratios compared to the rest of the population. A statistically significant correlation with the occurrence of insulin resistance and hyperprolactinemia was also confirmed.

The subpopulation with TSH ≤ 2.5 and negative ATA included 225 women (*n* = 225/367; 61.3%). The statistically significant lower value of fasting insulin concentrations at 60 and 120 min of the 75OGTT, significantly lower HOMA-IR value, significantly lower total cholesterol and triglycerides, and a significantly lower total cholesterol/HDL ratio, compared to the rest of the population studied, are noteworthy. A statistically significant lower percentage of insulin resistance, hyperprolactinemia and hypercholesterolemia were also confirmed. In the subgroup with TSH ≤ 2.5 and positive ATA (*n* = 28/367; 7.6%), apart from a statistically significantly higher concentration of SHBG, no significant correlation was found for any of the parameters tested. The presence of ATA, regardless of the TSH result (*n* = 44/367; 12.0%), was not associated with a significant effect on any of the analyzed parameters. There was no significant difference in the value of laboratory parameters, testosterone, LH, FSH, estradiol, prolactin, CRP, Ca-125, vitamin D concentrations, nor clinical parameters, age, body mass index (BMI), number of pregnancies, ovarian morphology, hypovitaminosis D, hyperandrogenism and mFerriman–Gallwey scale score, between subpopulations of women with PCOS with SCH, including ATA status and without thyroid dysfunction. The logistic regression model revealed a significantly increased risk of insulin resistance in the subpopulation of women with PCOS with TSH > 2.5 and negative ATA (OR 1.8, CI 1.13–2.85, *p* = 0.01 for HOMA-IR > 2.5; OR 1.81, CI 1.096–3.009, *p* = 0.02 for 120 min 75 OGTT insulin ≥ 100 µU/mL), as well as in the subpopulation of women with TSH > 2.5 regardless of ATA status (OR 1.91, CI 1.23–2.99, *p* = 0.004 for HOMA-IR > 2.5). In the latter group, the risk of hyperprolactinemia was also significantly increased (OR 2.04, CI 1.06–3.9, *p* = 0.03). Increased risk of insulin resistance (OR 1,63, CI 0.99–2.66, *p* = 0.0507) in the subgroup of women with PCOS with TSH > 2.5 regardless of ATA status, elevated risk of hypercholesterolemia (OR 1.7, CI 0.95–3.05, *p* = 0.07) and hyperprolactinemia (OR 1.84, CI 0.94–3.62, *p* = 0.07) in the subgroup of women with TSH > 2.5 and negative ATA and an increase in the likelihood of LH/FSH > 2 in the subpopulation of women with TSH > 2.5 and positive ATA (OR 4.43, CI 0.99–19.79, *p* = 0.051) was noticeable but did not reach statistical significance (Table 3). There was no significant association of any PCOS phenotype with SCH, nor with the presence of circulating ATA (Table 4). 

**Figure 1:**
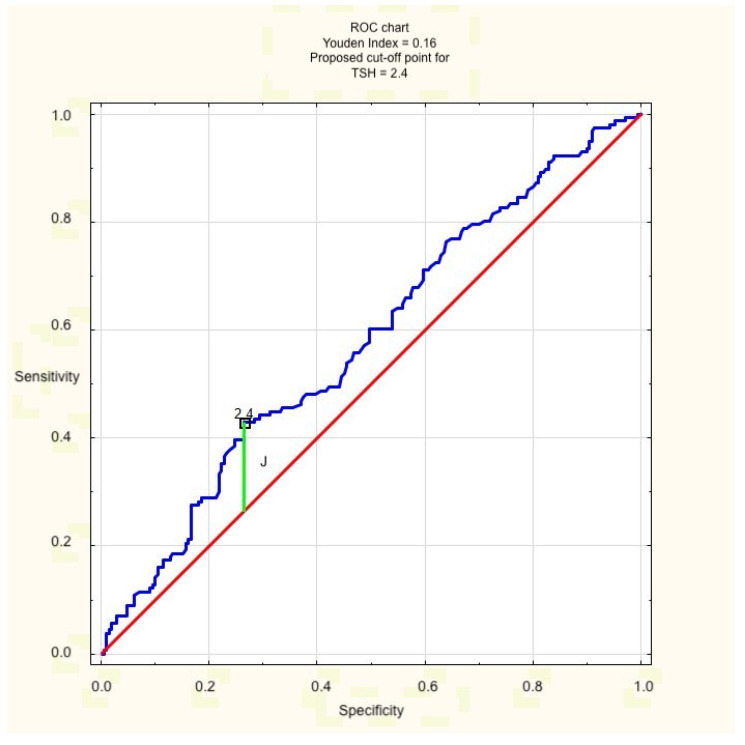
ROC curve for TSH cut-off point. J = Youden’s Index.

In the multivariate backward stepwise regression model, both hyperandrogenism (expressed as FAI > 5, but not elevated testosterone) and TSH > 2.5 turned out to be independent risk factors for insulin resistance, defined as HOMA-IR > 2.5 (W 60.75, *p* = 0.00, OR 6.44, CI 4.03–10.28 for FAI > 5; W 6.99, *p* = 0.008, OR 1.95, CI 1.188–3.185 for TSH > 2.5) in the examined population. The validity of the value of 2.5 as the cut-off point for TSH concentration was confirmed beforehand for the purpose of the study with the receiver operating characteristic (ROC) curve (Figure 1).

## 4. Discussion

The incidence of SCH in the study population (31.1%) was consistent with the results of other studies [13], while the incidence of circulating ATA (12%) and AIT (4.4%) was slightly lower [14,17], which may be due to the characteristics of the study population and the diagnostic criteria used. The study confirmed that TSH values > 2.5 corresponding to SCH were associated with the occurrence of significant metabolic disturbances, which was in line with the majority [13,24,25,35], but not all [36,37], of previous scientific reports, and the strongest relationship was found for hyperlipidemia and insulin resistance [38]. Interestingly, the coexistence of ATA in the subpopulation with TSH > 2.5 (AIT) did not seem to additionally affect the values of metabolic parameters and the effect of elevated TSH levels was even greater in the absence of ATA, which, however, requires further research and verification. What we are unable to interpret is the unexpected correlation of TSH and fT3, a parameter of less clinical significance than TSH and fT4 [39] in some subgroups of women with PCOS (Supplementary Table S1). However, this significantly elevated fT3 concentration was found in subpopulations of women with significantly elevated 120 min 75 OGTT insulin, which reflects the results of other studies [40], suggesting that fT3 could be an indicator of insulin resistance development. Despite an evidently higher incidence of insulin resistance and hyperlipidemia in the PCOS-SCH subpopulation, the expected associated higher testosterone level, higher FAI, higher mFG scale score, and higher prevalence of polycystic ovarian morphology have not been demonstrated, which is in line with the findings of other studies, with a few exceptions [41]. While the relationship between hyperandrogenism and insulin resistance, although not fully understood, is well established, the relationship between SCH and insulin resistance in PCOS is more ambiguous. In the examined population of women with newly diagnosed PCOS, SCH was confirmed to be an independent risk factor for insulin resistance. None of the PCOS phenotypes were found to be more frequent than others in the SCH population, which means that screening for hypothyroidism should be performed for all PCOS phenotypes. The results of the study also confirmed the fact that the diagnosis of SCH does not exclude the diagnosis of PCOS [42]. In the study population, phenotype D was recognized, as none of the women were diagnosed with overt hypothyroidism. No effect of SCH on fertility parameters (FSH and estradiol levels, number of pregnancies) was found, except for a significantly thinner endometrium and a higher LH/FSH ratio in the subgroup with TSH > 2.5 and positive ATA (AIT), which, however, are parameters of an uncertain diagnostic value. Although the subpopulation of women with TSH > 2.5 regardless of ATA status showed a higher risk of hyperprolactinemia, the value of prolactin concentration was not significantly higher than in the rest of the population. Since the literature does not provide sufficient evidence for an association between SCH and hyperprolactinemia, this result should be interpreted with caution [37]. Contrary to the reports from some of the previous studies [22,23,27], the mere presence of circulating ATA did not significantly influence the value of any parameter tested; thus, the more controversial relationship between AIT and PCOS has not been demonstrated, confirming observations from a few other studies [14], though they are few. The role of the immune system in the etiology of PCOS has been postulated for a long time [43], but so far, no cause–effect relationship or pathomechanism has been demonstrated. Systematic reviews indicated increased incidence of AIT in the PCOS population [17], but the role of antithyroid antibodies and the common ground of the two entities have yet to be established. Thus, high-normal TSH levels in women with PCOS require the diagnostics of insulin resistance and dyslipidemia in order to prevent potential adverse health effects in the future. The mechanism in which the elevated TSH concentration in euthyroid women with PCOS alters the results of metabolic parameters remains a target for future research.

## 5. Conclusions

The results of the study showed a clear correlation of SCH with metabolic, but not hormonal, parameters of women with PCOS. Women with PCOS, regardless of the phenotype, should be screened for hypothyroidism and screening for insulin resistance should be performed in women with SCH. Of all the parameters that are indicative of thyroid function, TSH was the most important. The potential effect of positive anti-thyroid antibodies was insignificant. The underlying pathomechanism remains a target for further research.

## Figures and Tables

**Table 1:** Study population characteristics regarding selected clinical, hormonal and metabolic parameters (N = 367).

Variable	Value *						
	Mean	SD	Q1	Median	Q3	Minimum	Maximum
Age	24.28	4.99	21.00	24.00	27.00	16.00	44.00
Cycle day	65.53	133.97	13.00	24.00	57.00	2.00	1891.00
Endometrium thickness	6.91	2.95	5.00	6.75	9.00	1.00	19.00
BMI [kg/m^2^]	25.47	6.61	20.32	23.15	29.97	16.18	54.08
Fasting blood glucose [mmol/L]	4.95	0.52	4.62	4.90	5.17	3.69	8.63
Glucose 60 min OGTT [mmol/L]	7.05	2.10	5.51	6.76	8.36	2.98	13.30
Glucose 120 min OGTT [mmol/L]	5.78	1.47	4.82	5.72	6.63	0.90	10.90
Fasting blood insulin [µU/mL]	13.11	10.49	6.88	9.88	16.01	1.88	114.00
Insulin 60 min OGTT [µU/mL]	111.91	88.17	52.25	86.36	144.30	3.82	555.00
Insulin 120 min OGTT [µU/mL]	86.76	84.89	37.80	58.00	99.80	4.72	735.00
HOMA-IR	2.97	2.59	1.47	2.15	3.66	0.36	25.99
FAI	5.26	4.14	2.46	4.18	6.95	0.10	39.20
Total Cholesterol [mmol/L]	4.50	0.77	4.00	4.40	5.00	3.00	8.00
HDL Cholesterol [mmol/L]	1.62	0.44	1.32	1.53	1.87	0.66	3.60
Total Cholesterol/HDL	2.97	0.95	2.25	2.74	3.50	1.32	7.29
LDL Cholesterol [mmol/L]	2.41	0.68	2.00	2.30	2.80	0.70	5.50
LDL/HDL	1.62	0.67	1.11	1.48	2.05	0.24	4.06
Triglycerides [mmol/L]	1.06	0.55	0.69	0.89	1.23	0.34	3.52
TSH [mU/L]	2.17	1.11	1.46	1.98	2.67	0.28	9.67
fT3 [pmol/L]	5.26	0.77	4.74	5.17	5.75	3.12	7.52
fT4 [pmol/L]	15.40	2.40	13.70	15.20	16.62	12.00	24.83
anti-TPO [IU/mL]	22.98	42.42	9.45	11.72	16.70	5.00	374.00
anti-TG [IU/mL]	50.19	176.10	10.00	13.90	18.30	10.00	2533.00
Prolactin [µU/mL]	334.41	156.15	235.00	308.00	405.00	86.45	1427.00
SHBG [nmol/L]	50.90	33.12	27.60	43.60	67.60	8.70	355.00
Testosterone [nmol/L]	1.87	0.69	1.43	1.82	2.24	0.29	4.62
LH [mU/mL]	13.43	8.64	7.55	11.70	16.90	0.78	52.16
FSH [mU/mL]	5.61	5.16	4.07	5.48	6.64	0.87	14.80
LH/FSH ratio	2.5	1.27	1.60	2.26	3.09	0.40	9.14
Estradiol [ng/mL]	335.57	288.19	169.40	220.50	375.00	18.40	2070.00
CRP	2.65	3.92	1.00	1.00	2.51	0.99	37.30
Ca-125	12.40	6.79	8.10	10.80	14.50	4.00	77.30
Vitamin D [ng/mL]	24.43	11.40	16.73	23.40	28.60	5.12	129.00
mFerriman–Gallwey scale score	10.83	6.75	6.00	10.00	15.00	0.00	30.00

* Reference values: Fasting blood glucose < 5.6 mmol/L, fasting blood insulin < 13 uU/mL, glucose 120 min. OGTT < 11.0 mmol/L, HOMA-IR < 2.5, FAI < 5, Total Cholesterol 3.2–5.2 mmol/L, HDL Cholesterol > 1.2 mmol/L, LDL Cholesterol < 3.4 mmol/L, Triglycerides < 2.26 mmol/L, TSH 0.27–4.2 uIU/mL, fT3 3.1–6.8 pmol/L, fT4 12.0–22.0 pmol/L, anti-TPO < 34.0 IU/mL, anti-TG < 115.0 IU/mL, Prolactin 102–496 uIU/mL, SHBG 26.1–110 nmol/L, testosterone 0.29–1.67 nmol/L, LH 2.4–12.6 mIU/mL, FSH 3.5–12.5 mIU/mL, estradiol 46–607 pmol/L, CRP < 5.00 mg/L, Ca-125 < 35.0 U/mL, vitamin D 30.00–80.00 ng/mL, mFerriman–Gallwey scale score < 8.

**Table 2:** Study population characteristics in relation to selected qualitative variables (N = 367).

Variable	Value	
	*n*	%
Pregnancy	28	7.6
Polycystic ovarian morphology	239	65.1
Insulin resistance	156	42.5
Hyperandrogenism [FAI]	146	39.8
Hypercholesterolemia	60	16.3
High LDL	26	7.1
Hypertriglyceridemia	20	5.4
Hyperprolactinemia	41	11.2
Hypovitaminosis D	284	77.4

**Table 3:** Logistic regression model results for selected parameters in subpopulations of women with PCOS.

**Insulin resistance: HOMA-IR > 2.5**			
**Variable**	**OR**	**CI**	***p***
TSH > 2.5 and negative anti-thyroid antibodies	1.8	1.13–2.85	0.01
TSH > 2.5 and positive anti-thyroid antibodies	1.78	0.65–4.9	0.26
TSH ≤ 2.5 and positive antibodies	0.52	0.22–1.2	0.12
TSH > 2.5 regardless of anti-thyroid antibodies status	1.91	1.23–2.99	0.004
Presence of anti-thyroid antibodies regardless of TSH	0.83	0.43–1.58	0.58
**Insulin resistance: fasting insulin ≥ 13** **µU/mL**			
**Variable**	**OR**	**CI**	***p***
TSH > 2.5 and negative anti-thyroid antibodies	1.40	0.87–2.25	0.16
TSH > 2.5 and positive anti-thyroid antibodies	1.97	0.72–5.39	0.18
TSH ≤ 2.5 and positive antibodies	0.89	0.39–2.04	0.79
TSH > 2.5 regardless of anti-thyroid antibodies status	1.56	0.99–2.47	0.054
Presence of anti-thyroid antibodies regardless of TSH	1.23	0.64–2.36	0.52
**Insulin resistance: insulin at 120 min. 75 OGTT ≥ 100** **µU/mL**			
**Variable**	**OR**	**CI**	***p***
TSH > 2.5 and negative anti-thyroid antibodies	1.81	1.096–3.009	0.02
TSH > 2.5 and positive anti-thyroid antibodies	0.66	0.18–2.4	0.53
TSH ≤ 2.5 and positive antibodies	0.98	0.40–2.38	0.96
TSH > 2.5 regardless of anti-thyroid antibodies status	1.63	0.99–2.66	0.0507
Presence of anti-thyroid antibodies regardless of TSH	0.85	0.40–1.79	0.67
**Hyperandrogenism**			
**Variable**	**OR**	**CI**	***p***
TSH > 2.5 and negative anti-thyroid antibodies	1.27	0.8–2.02	0.31
TSH > 2.5 and positive anti-thyroid antibodies	1.19	0.43–3.26	0.74
TSH ≤ 2.5 and positive antibodies	0.98	0.44–2.15	0.95
TSH > 2.5 regardless of anti-thyroid antibodies status	1.28	0.82–2.01	0.26
Presence of anti-thyroid antibodies regardless of TSH	1.055	0.55–2.00	0.87
**Hypercholesterolemia**			
**Variable**	**OR**	**CI**	***p***
TSH > 2.5 and negative anti-thyroid antibodies	1.7	0.95–3.05	0.07
TSH > 2.5 and positive anti-thyroid antibodies	1.19	0.33–4.31	0.79
TSH ≤ 2.5 and positive antibodies	1.44	0.56–3.72	0.45
TSH > 2.5 regardless of anti-thyroid antibodies status	1.69	0.96–2.99	0.06
Presence of anti-thyroid antibodies regardless of TSH	1.37	0.62–3.02	0.43
**Hypertriglyceridemia**			
**Variable**	**OR**	**CI**	***p***
TSH > 2.5 and negative anti-thyroid antibodies	1.47	0.57–3.8	0.42
TSH > 2.5 and positive anti-thyroid antibodies	1.16	0.15–9.28	0.88
TSH ≤ 2.5 and positive antibodies	1.37	0.3–6.24	0.68
TSH > 2.5 regardless of anti-thyroid antibodies status	1.47	0.58–3.71	0.4
Presence of anti-thyroid antibodies regardless of TSH	1.31	0.37–4.69	0.67
**Hyperprolactinemia**			
**Variable**	**OR**	**CI**	***p***
TSH > 2.5 and negative anti-thyroid antibodies	1.84	0.94–3.62	0.07
TSH > 2.5 and positive anti-thyroid antibodies	1.9	0.52–6.97	0.33
TSH ≤ 2.5 and positive antibodies	0.95	0.27–3.3	0.93
TSH > 2.5 regardless of anti-thyroid antibodies status	2.04	1.06–3.9	0.03
Presence of anti-thyroid antibodies regardless of TSH	1.29	0.51—3.3	0.58
**LH/FSH > 2**			
**Variable**	**OR**	**CI**	***p***
TSH > 2.5 and negative anti-thyroid antibodies	1.17	0.72–1.88	0.52
TSH > 2.5 and positive anti-thyroid antibodies	4.43	0.99–19.79	0.051
TSH ≤ 2.5 and positive antibodies	0.67	0.31–1.46	0.31
TSH > 2.5 regardless of anti-thyroid antibodies status	1.44	0.9–2.28	0.12
Presence of anti-thyroid antibodies regardless of TSH	1.18	0.6–2.3	0.6

**Table 4:** PCOS phenotypes in the studied subpopulations of PCOS women.

**TSH > 2.5 and positive anti-thyroid antibodies (*n* = 16, N = 367)**			
**Variable**	**% *n***	**% N-*n***	***p***
Phenotype A	50% (8)	52% (182)	0.88
Phenotype B	37% (6)	34% (121)	0.80
Phenotype C	6.5% (1)	5% (17)	0.80
Phenotype D	6.5% (1)	9% (31)	0.7
**TSH > 2.5 and negative anti-thyroid antibodies (*n* = 98, N = 367)**			
**Variable**	**% *n***	**% N-*n***	***p***
Phenotype A	52% (52)	51% (138)	0.95
Phenotype B	34% (34)	35% (93)	0.88
Phenotype C	5% (5)	5% (13)	0.95
Phenotype D	9% (9)	9% (23)	0.9
**TSH > 2.5 regardless anti-thyroid antibodies (*n* = 114, N = 367)**			
**Variable**	**% *n***	**% N-*n***	***p***
Phenotype A	52% (60)	52% (130)	0.99
Phenotype B	35% (40)	35% (87)	0.95
Phenotype C	5% (6)	5% (12)	0.93
Phenotype D	8% (9)	8% (22)	0.96
**TSH ≤ 2.5 and positive anti-thyroid antibodies (*n* = 28, N = 367)**			
**Variable**	**% *n***	**% N-*n***	***p***
Phenotype A	50% (14)	52% (176)	0.84
Phenotype B	32% (9)	35% (118)	0.77
Phenotype C	7% (2)	5% (16)	0.59
Phenotype D	11% (3)	8% (29)	0.7
**TSH ≤ 2.5 and negative anti-thyroid antibodies (*n* = 225, N = 367)**			
**Variable**	**% *n***	**% N-*n***	***p***
Phenotype A	52% (116)	51.5% (74)	0.9
Phenotype B	35% (78)	34% (49)	0.85
Phenotype C	4.5% (10)	5.5% (8)	0.64
Phenotype D	8.5% (19)	9% (13)	0.86
**Presence of anti-thyroid antibodies regardless of TSH (*n* = 44, N = 367)**			
**Variable**	**% *n***	**% N-*n***	***p***
Phenotype A	50% (22)	52% (168)	0.80
Phenotype B	34% (15)	34% (112)	0.93
Phenotype C	7% (3)	5% (15)	0.54
Phenotype D	9% (4)	9% (28)	0.92

## Data Availability

Data available on request.

## Supplementary Materials

The following supporting information can be downloaded at: https://www.mdpi.com/article/10.3390/jcm11061547/s1, Table S1. The characteristics of the subpopulations in terms of TSH values and ATA status in relation to selected quantitative parameters. Table S2. The characteristics of the subpopulations in terms of TSH values and ATA status in relation to selected qualitative parameters.

## References

[1] Teede H.J., Misso M.L., Costello M.F., Dokras A., Laven J., Moran L., Piltonen T., Norman R.J. (2018). International PCOS Network. Recommendations from the international evidence-based guideline for the assessment and management of polycystic ovary syndrome. Hum. Reprod..

[2] Chang S., Dunaif A. (2021). Diagnosis of Polycystic Ovary Syndrome: Which Criteria to Use and When?. Endocrinol. Metab. Clin. N. Am..

[3] Rotterdam ESHRE/ASRM-Sponsored PCOS Consensus Workshop Group (2004). Revised 2003 consensus on diagnostic criteria and long-term health risks related to polycystic ovary syndrome. Fertil. Steril..

[4] Vink J.M., Sadrzadeh S., Lambalk C.B., Boomsma D.I. (2006). Heritability of Polycystic Ovary Syndrome in a Dutch Twin-Family Study. J. Clin. Endocrinol. Metab..

[5] Hiam D., Simar D., Laker R., Altıntaş A., Gibson-Helm M., Fletcher E., Moreno-Asso A., Trewin A.J., Barres R., Stepto N.K. (2019). Epigenetic Reprogramming of Immune Cells in Women with PCOS Impact Genes Controlling Reproductive Function. J. Clin. Endocrinol. Metab..

[6] Mimouni N.E.H., Paiva I., Barbotin A.-L., Timzoura F.E., Plassard D., Le Gras S., Ternier G., Pigny P., Catteau-Jonard S., Simon V. (2021). Polycystic ovary syndrome is transmitted via a transgenerational epigenetic process. Cell Metab..

[7] Dabravolski S., Nikiforov N., Eid A., Nedosugova L., Starodubova A., Popkova T., Bezsonov E., Orekhov A. (2021). Mitochondrial Dysfunction and Chronic Inflammation in Polycystic Ovary Syndrome. Int. J. Mol. Sci..

[8] Sanchez-Garrido M.A., Tena-Sempere M. (2020). Metabolic dysfunction in polycystic ovary syndrome: Pathogenic role of androgen excess and potential therapeutic strategies. Mol. Metab..

[9] Palioura E., Diamanti-Kandarakis E. (2015). Polycystic ovary syndrome (PCOS) and endocrine disrupting chemicals (EDCs). Rev. Endocr. Metab. Disord..

[10] Wild R.A., Carmina E., Diamanti-Kandarakis E., Dokras A., Escobar-Morreale H., Futterweit W., Lobo R., Norman R., Talbott E., Dumesic D.A. (2010). Assessment of Cardiovascular Risk and Prevention of Cardiovascular Disease in Women with the Polycystic Ovary Syndrome: A Consensus Statement by the Androgen Excess and Polycystic Ovary Syndrome (AE-PCOS) Society. J. Clin. Endocrinol. Metab..

[11] Barry J., Azizia M.M., Hardiman P.J. (2014). Risk of endometrial, ovarian and breast cancer in women with polycystic ovary syndrome: A systematic review and meta-analysis. Hum. Reprod. Update.

[12] Glintborg D., Rubin K.H., Nybo M., Abrahamsen B., Andersen M. (2019). Increased risk of thyroid disease in Danish women with polycystic ovary syndrome: A cohort study. Endocr. Connect..

[13] Fatima M., Amjad S., Ali Sr H.S., Ahmed T., Khan S., Raza M., Inam M. (2020). Correlation of Subclinical Hypothyroidism With Polycystic Ovary Syndrome (PCOS). Cureus.

[14] Garelli S., Masiero S., Plebani M., Chen S., Furmaniak J., Armanini D., Betterle C. (2013). High prevalence of chronic thyroiditis in patients with polycystic ovary syndrome. Eur. J. Obstet. Gynecol. Reprod. Biol..

[15] Zeber-Lubecka N., Hennig E.E. (2021). Genetic Susceptibility to Joint Occurrence of Polycystic Ovary Syndrome and Hashimoto’s Thyroiditis: How Far Is Our Understanding?. Front. Immunol..

[16] Tuten A., Hatipoglu E., Oncul M., Imamoglu M., Acikgoz A.S., Yilmaz N., Ozcil M.D., Kaya B., Misirlioglu A.M., Sahmay S. (2014). Evaluation of ovarian reserve in Hashimoto’s thyroiditis. Gynecol. Endocrinol..

[17] Romitti M., Fabris V.C., Ziegelmann P.K., Maia A.L., Spritzer P.M. (2018). Association between PCOS and autoimmune thyroid disease: A systematic review and meta-analysis. Endocr. Connect..

[18] Artini P.G., Uccelli A., Papini F., Simi G., Di Berardino O.M., Ruggiero M., Cela V. (2012). Infertility and pregnancy loss in euthyroid women with thyroid autoimmunity. Gynecol. Endocrinol..

[19] Korevaar T.I.M., Derakhshan A., Taylor P.N., Meima M., Chen L., Bliddal S., Carty D.M., Meems M., Vaidya B., Consortium on Thyroid and Pregnancy—Study Group on Preterm Birth (2019). Association of Thyroid Function Test Abnormalities and Thyroid Autoimmunity With Preterm Birth: A Systematic Review and Meta-analysis. JAMA.

[20] Ott J., Aust S., Kurz C., Nouri K., Wirth S., Huber J.C., Mayerhofer K. (2010). Elevated antithyroid peroxidase antibodies indicating Hashimoto’s thyroiditis are associated with the treatment response in infertile women with polycystic ovary syndrome. Fertil. Steril..

[21] Poppe K., Bisschop P., Fugazzola L., Minziori G., Unuane D., Weghofer A. (2021). 2021 European Thyroid Association Guideline on Thyroid Disorders prior to and during Assisted Reproduction. Eur. Thyroid J..

[22] Zhao H., Zhang Y., Ye J., Wei H., Huang Z., Ning X., Fu X. (2021). A Comparative Study on Insulin Secretion, Insulin Resistance and Thyroid Function in Patients with Polycystic Ovary Syndrome with and without Hashimoto’s Thyroiditis. Diabetes Metab. Syndr. Obes..

[23] Ho C.-W., Chen H.-H., Hsieh M.-C., Chen C.-C., Hsu S.-P., Yip H.-T., Kao C.-H. (2020). Increased Risk of Polycystic Ovary Syndrome and It’s Comorbidities in Women with Autoimmune Thyroid Disease. Int. J. Environ. Res. Public Health.

[24] Bedaiwy M.A., Abdel-Rahman M.Y., Tan J., Abdelhafez F.F., Abdelkareem A.O., Henry D., Lisonkova S., Hurd W.W., Liu J.H. (2018). Clinical, Hormonal, and Metabolic Parameters in Women with Subclinical Hypothyroidism and Polycystic Ovary Syndrome: A Cross-Sectional Study. J. Women’s Health.

[25] Kalra S., Aggarwal S., Khandelwal D. (2021). Thyroid Dysfunction and Dysmetabolic Syndrome: The Need for Enhanced Thyrovigilance Strategies. Int. J. Endocrinol..

[26] Adamska A., Łebkowska A., Krentowska A., Hryniewicka J., Adamski M., Leśniewska M., Polak A.M., Kowalska I. (2020). Ovarian Reserve and Serum Concentration of Thyroid Peroxidase Antibodies in Euthyroid Women With Different Polycystic Ovary Syndrome Phenotypes. Front. Endocrinol..

[27] Kim J.J., Yoon J.W., Kim M.J., Kim S.M., Hwang K.R., Choi Y.M. (2022). Thyroid autoimmunity markers in women with polycystic ovary syndrome and controls. Hum. Fertil..

[28] Trakakis E., Pergialiotis V., Hatziagelaki E., Panagopoulos P., Salloum I., Papantoniou N. (2017). Subclinical hypothyroidism does not influence the metabolic and hormonal profile of women with PCOS. Horm. Mol. Biol. Clin. Investig..

[29] Pergialiotis V., Konstantopoulos P., Prodromidou A., Florou V., Papantoniou N., Perrea D.N. (2017). MANAGEMENT OF ENDOCRINE DISEASE: The impact of subclinical hypothyroidism on anthropometric characteristics, lipid, glucose and hormonal profile of PCOS patients: A systematic review and meta-analysis. Eur. J. Endocrinol..

[30] Alexander E.K., Pearce E.N., Brent G.A., Brown R.S., Chen H., Dosiou C., Grobman W.A., Laurberg P., Lazarus J.H., Mandel S.J. (2017). 2017 Guidelines of the American Thyroid Association for the Diagnosis and Management of Thyroid Disease During Pregnancy and the Postpartum. Thyroid.

[31] Martin K.A., Anderson R.R., Chang R.J., Ehrmann D.A., Lobo R.A., Murad M.H., Pugeat M.M., Rosenfield R.L. (2018). Evaluation and Treatment of Hirsutism in Premenopausal Women: An Endocrine Society Clinical Practice Guideline. J. Clin. Endocrinol. Metab..

[32] Salley K.E., Wickham E.P., Cheang K.I., Essah P.A., Karjane N.W., Nestler J.E. (2007). Glucose intolerance in polycystic ovary syndrome—A position statement of the Androgen Excess Society. J. Clin. Endocrinol. Metab..

[33] Carmina E., Lobo R.A. (2004). Use of fasting blood to assess the prevalence of insulin resistance in women with polycystic ovary syndrome. Fertil. Steril..

[34] Matthews D.R., Hosker J.P., Rudenski A.S., Naylor B.A., Treacher D.F., Turner R.C. (1985). Homeostasis model assessment: Insulin resistance and beta-cell function from fasting plasma glucose and insulin concentrations in man. Diabetologia.

[35] Lu Y.-H., Xia Z.-L., Ma Y.-Y., Chen H.-J., Yan L.-P., Xu H.-F. (2016). Subclinical hypothyroidism is associated with metabolic syndrome and clomiphene citrate resistance in women with polycystic ovary syndrome. Gynecol. Endocrinol..

[36] Ganie M.A., Laway B.A., Wani T.A., Zargar M.A., Nisar S., Ahamed F., Khurana M., Ahmed S. (2011). Association of subclinical hypothyroidism and phenotype, insulin resistance, and lipid parameters in young women with polycystic ovary syndrome. Fertil. Steril..

[37] Benetti-Pinto C.L., Piccolo V.R.S.B., Garmes H.M., Juliato C.R.T. (2013). Subclinical hypothyroidism in young women with polycystic ovary syndrome: An analysis of clinical, hormonal, and metabolic parameters. Fertil. Steril..

[38] Mueller A., Schofl C., Dittrich R., Cupisti S., Oppelt P., Schild R., Beckmann M., Haberle L. (2009). Thyroid-stimulating hormone is associated with insulin resistance independently of body mass index and age in women with polycystic ovary syndrome. Hum. Reprod..

[39] Fitzgerald S.P., Bean N.G., Falhammar H., Tuke J. (2020). Clinical Parameters Are More Likely to Be Associated with Thyroid Hormone Levels than with Thyrotropin Levels: A Systematic Review and Meta-Analysis. Thyroid.

[40] Štěpánek L., Horáková D., Štěpánek L., Janout V., Janoutová J., Bouchalová K., Martiník K. (2021). Free triiodothyronine/free thyroxine (FT3/FT4) ratio is strongly associated with insulin resistance in euthyroid and hypothyroid adults: A cross-sectional study. Endokrynol. Polska.

[41] Cai J., Zhang Y., Wang Y., Li S., Wang L., Zheng J., Jiang Y., Dong Y., Zhou H., Hu Y. (2019). High Thyroid Stimulating Hormone Level Is Associated With Hyperandrogenism in Euthyroid Polycystic Ovary Syndrome (PCOS) Women, Independent of Age, BMI, and Thyroid Autoimmunity: A Cross-Sectional Analysis. Front. Endocrinol..

[42] de-Medeiros S.F., Yamamoto M.M.W., de-Medeiros M.A.S., Barbosa J.S., Norman R.J. (2017). Should Subclinical Hypothyroidism Be an Exclusion Criterion for the Diagnosis of Polycystic Ovary Syndrome?. J. Reprod. Infertil..

[43] Hefler-Frischmuth K., Walch K., Huebl W., Baumuehlner K., Tempfer C., Hefler L. (2010). Serologic markers of autoimmunity in women with polycystic ovary syndrome. Fertil. Steril..

