# Supplementary material for: Association of Subclinical Hypothyroidism with Present and Absent Anti-Thyroid Antibodies with PCOS Phenotypes and Metabolic Profile
Source: J Clin Med. 2022 Mar 11;11(6):1547. doi: 10.3390/jcm11061547 (PMC8948753; doi:10.3390/jcm11061547)
Supplement: Supplementary file 1 [file jcm-11-01547-s001.zip › jcm-1635080-supplementary.pdf]

Supplementary Table S1. The characteristics of the subpopulations in terms of TSH values and ATA status in relation to selected quantitative parameters.

Subpopulation	Value																													
	**TSH >2.5 and positive ATA (*n*=16, N=367)**					**TSH >2.5 and negative ATA (*n*=98, N=367)**					**TSH >2.5 regardless ATA status (*n*=114, N=367)**					**TSH ≤2.5 and negative ATA (*n*=225, N=367)**					**TSH ≤2.5 and positive ATA (*n*=28, N=367)**					**ATA positive regardless of the TSH result (*n*=44, N=367)**				
**Variable**	Mean N-*n*	Mean *n*	p	SD N-*n*	SD *n*	Mean N-*n*	Mean *n*	*p*	SD N-*n*	SD *n*	Mean N-*n*	Mean *n*	*p*	SD N-*n*	SD *n*	Mean *n*	Mean N-*n*	*p*	SD *n*	SD N-*n*	Mean N-*n*	Mean *n*	*p*	SD N-*n*	SD *n*	Mean N-*n*	Mean *n*	*p*	SD N-*n*	SD *n*
Age	24.24	25.19	0.46	4.89	7.07	24.15	24.63	0.42	4.79	5.51	24.09	24.71	0.27	4.6	5.72	24.13	24.51	0.48	4.57	5.59	24.33	23.71	0.53	4.99	5.07	24.29	24.25	0.96	4.88	5.84
Endometrium thickness	6.98	5.45	0.05	2.97	2.03	6.77	7.31	0.13	2.83	3.23	6.85	7.05	0.56	2.86	3.15	6.89	6.95	0.86	2.94	2.98	6.93	6.67	0.66	3	2.25	7	6.24	0.11	3.02	2.23
BMI	25.45	26.12	0.82	6.66	5.02	25.37	25.72	0.76	6.69	6.48	25.33	25.76	0.7	6.77	6.31	25.37	25.61	0.83	6.67	6.56	25.44	25.79	0.86	6.52	7.87	25.42	25.88	0.78	6.58	7.05
Fasting blood glucose [mmol/L]	4.95	4.96	0.9	0.52	0.38	4.92	5.02	0.09	0.54	0.44	4.92	5.01	0.09	0.55	0.43	4.92	4.99	0.24	0.56	0.44	4.95	4.88	0.5	0.52	0.49	4.95	4.91	0.64	0.52	0.45
OGTT 60 min. [mmol/L]	7.06	6.91	0.78	2.09	2.35	6.92	7.41	0.05	2.16	1.88	6.92	7.34	0.08	2.16	1.95	6.93	7.24	0.16	2.17	1.98	7.06	7.01	0.92	2.11	2.07	7.06	6.98	0.8	2.1	2.15
OGTT 120 min. [mmol/L]	5.78	5.8	0.97	1.48	1.27	5.68	6.06	0.03	1.41	1.58	5.68	6.03	0.03	1.42	1.54	5.68	5.94	0.1	1.44	1.49	5.79	5.68	0.7	1.48	1.28	5.79	5.72	0.77	1.49	1.26
Fasting blood insulin [µU/mL]	12.84	19	0.02	9.15	26.2	12.23	15.5	0.01	10.05	11.31	11.79	15.99	0	7.94	14.23	11.97	14.86	0.01	8.15	13.16	13.31	10.59	0.2	10.74	6.19	13.03	13.72	0.68	9.34	16.91
Insulin 60 min. OGTT [µU/mL]	110.54	141.6	0.17	84.06	153.94	104.7	131.47	0.01	82.31	100.28	102.34	132.89	0	75.42	108.58	101.69	127.71	0.01	73.64	105.16	111.9	112	1	88.1	90.77	110.42	123.02	0.38	83.62	117.36
Insulin 120 min. OGTT [µU/mL]	86.86	84.54	0.91	84.81	89.4	75.64	117.05	0	65.94	117.7	75.08	112.49	0	64.35	114.38	75.46	104.3	0	63.42	108.19	87.62	76.03	0.5	85.78	73.42	87.77	79.2	0.53	85.74	78.79
HOMA-IR	2.91	4.28	0.04	2.32	6.02	2.76	3.51	0.01	2.54	2.67	2.67	3.62	0	2.13	3.31	2.71	3.35	0.02	2.2	3.07	3.01	2.35	0.2	2.66	1.49	2.95	3.07	0.78	2.38	3.9
FAI	5.2	6.57	0.2	4.06	5.54	4.99	6.02	0.04	3.55	5.39	4.89	6.09	0.01	3.37	5.39	4.99	5.69	0.11	3.46	5	5.35	4.24	0.17	4.23	2.65	5.29	5.09	0.77	4.16	4.05
Total Cholesterol [mmol/L]	4.5	4.65	0.43	0.77	0.77	4.45	4.64	0.04	0.75	0.81	4.44	4.64	0.02	0.74	0.8	4.44	4.61	0.04	0.75	0.79	4.5	4.51	0.93	0.77	0.75	4.49	4.56	0.57	0.77	0.75
HDL Cholesterol [mmol/L]	1.62	1.59	0.83	0.44	0.39	1.64	1.56	0.15	0.43	0.47	1.64	1.57	0.14	0.43	0.46	1.63	1.59	0.39	0.42	0.46	1.62	1.62	0.93	0.44	0.42	1.62	1.61	0.95	0.44	0.4
Total Cholesterol/HDL	2.97	3.08	0.64	0.95	0.93	2.9	3.19	0.01	0.92	0.99	2.88	3.18	0.01	0.92	0.98	2.89	3.11	0.03	0.93	0.97	2.98	2.93	0.80	0.95	0.84	2.97	2.99	0.93	0.96	0.87
LDL Cholesterol [mmol/L]	2.41	2.29	0.49	0.68	0.58	2.37	2.53	0.04	0.67	0.68	2.37	2.5	0.09	0.68	0.67	2.37	2.46	0.22	0.67	0.68	2.41	2.42	0.94	0.68	0.65	2.41	2.37	0.71	0.68	0.63
LDL/HDL	1.62	1.53	0.59	0.67	0.51	1.56	1.77	0.01	0.65	0.7	1.57	1.73	0.03	0.66	0.68	1.57	1.7	0.08	0.64	0.7	1.62	1.62	0.96	0.66	0.72	1.62	1.59	0.77	0.67	0.65
Triglycerides [mmol/L]	1.04	1.29	0.08	0.54	0.75	1.01	1.18	0.01	0.52	0.59	0.99	1.19	0	0.5	0.62	0.99	1.16	0	0.5	0.61	1.06	1.06	0.96	0.55	0.57	1.04	1.14	0.25	0.53	0.64
TSH [mU/L]	2.11	3.59	0	1.06	1.18	1.74	3.37	0	0.73	1.09	1.62	3.38	0	0.49	1.1	1.63	3.02	0	0.49	1.25	2.23	1.54	0	1.13	0.52	2.16	2.28	0.48	1.08	1.29
fT3 [pmol/L]	5.25	5.47	0.27	0.76	0.98	5.14	5.58	0	0.72	0.82	5.12	5.56	0	0.7	0.84	5.11	5.48	0	0.7	0.83	5.27	5.16	0.47	0.78	0.72	5.26	5.27	0.92	0.77	0.82
fT4 [pmol/L]	15.4	15.43	0.96	2.41	2.08	15.34	15.57	0.41	2.35	2.52	15.34	15.53	0.48	2.38	2.45	15.39	15.41	0.93	2.42	2.37	15.44	14.93	0.28	2.43	1.96	15.44	15.11	0.4	2.45	2
anti-TPO [IU/mL]	19.92	90.09	0	37.8	74.42	26.45	13.46	0.01	49.02	4.86	22.53	23.96	0.76	44.35	38.06	12.54	39.15	0	4.71	64.32	16.45	102.08	0	23.17	103.16	12.8	97.72	0	4.76	93

anti-TG [IU/mL]	34.83	387.11	0	107.81	599.11	61.99	17.81	0.03	204.27	16.97	41.67	68.63	0.17	126.47	251.85	15.21	104.36	0	10.37	272.66	33.49	252.39	0	149.32	308.84	15.97	301.38	0	12.75	435.2
Prolactin [µU/mL]	333.43	355.98	0.57	156	163.18	329.9	346.79	0.36	158.33	150.13	328.25	348.08	0.26	158.2	151.3	325.9	347.58	0.19	160.88	148.13	333.62	343.95	0.74	157.41	142.31	332.51	348.32	0.53	157.3	148.46
SHBG [nmol/L]	51.21	44.26	0.41	33.36	27.38	50.8	51.19	0.92	32.24	35.58	51.21	50.21	0.79	32.53	34.52	49.45	53.16	0.29	26.28	41.55	49.88	63.27	0.04	29.43	62.08	50.16	56.36	0.24	29.54	52.61
Testosterone [nmol/L]	1.87	1.94	0.71	0.69	0.68	1.85	1.94	0.25	0.67	0.73	1.84	1.94	0.21	0.67	0.72	1.84	1.92	0.25	0.66	0.73	1.87	1.87	0.95	0.68	0.81	1.87	1.89	0.86	0.68	0.76
LH [mU/mL]	13.3	16.4	0.16	8.76	4.62	13.11	14.32	0.24	8.3	9.51	12.9	14.61	0.08	8.44	9	13.09	13.97	0.34	8.5	8.87	13.6	11.38	0.19	8.66	8.27	13.47	13.21	0.85	8.8	7.5
FSH [mU/mL]	5.62	5.55	0.96	5.27	1.63	5.63	5.55	0.89	5.93	1.79	5.64	5.55	0.88	6.11	1.76	5.7	5.48	0.69	6.45	1.88	5.67	4.89	0.44	5.34	1.99	5.68	5.13	0.51	5.46	1.88
LH/FSH ratio	2.46	3.24	0.02	1.25	1.36	2.49	2.53	0.78	1.3	1.19	2.44	2.63	0.18	1.28	1.23	2.47	2.54	0.63	1.29	1.24	2.52	2.23	0.25	1.27	1.23	2.48	2.6	0.56	1.26	1.35
Estradiol [ng/mL]	336.27	320.26	0.83	289.07	276.38	343.27	314.42	0.4	296.22	265.17	344.73	315.24	0.37	297.89	265.52	343.03	324.02	0.54	281.78	298.47	333.01	366.53	0.55	275.6	417	333.64	349.71	0.73	275.98	369.24
CRP [mg/L]	2.66	2.45	0.84	3.99	1.84	2.56	2.88	0.49	4.05	3.54	2.57	2.82	0.57	4.15	3.35	2.47	2.92	0.28	3.57	4.4	2.58	3.44	0.26	3.49	7.43	2.59	3.08	0.43	3.55	6.01
CA-125 [U/mL]	12.43	11.73	0.69	6.85	5.48	12.27	12.74	0.56	6.94	6.38	12.31	12.6	0.7	7.03	6.25	12.35	12.47	0.88	7.35	5.85	12.42	12.08	0.8	6.97	4.08	12.46	11.96	0.65	7.04	4.58
Vitamin D [ng/mL]	24.42	24.52	0.97	11.16	16.26	24.45	24.36	0.95	10.41	13.82	24.44	24.38	0.96	9.98	14.11	24.6	24.16	0.72	10.05	13.26	24.48	23.77	0.75	11.54	9.71	24.48	24.04	0.81	11.29	12.31
mFerriman-Gallwey scale	10.86	10.08	0.68	6.73	7.44	10.74	11.06	0.69	6.48	7.47	10.78	10.94	0.83	6.44	7.43	10.94	10.66	0.71	6.39	7.31	10.94	9.54	0.29	6.72	7.07	10.98	9.71	0.26	6.7	7.1

Supplementary Table S2. The characteristics of the subpopulations in terms of TSH values and ATA status in relation to selected qualitative parameters.

Value					
TSH >2.5 and positive ATA (*n*=16, N=367)					
Variable	*p*	*n*	N-*n*	%*n*	% (N-*n*)
Pregnancy	0.82	16	351	6.3% (*n*=1)	7.7% (27)
Polycystic ovarian morphology	0.82	16	351	62.5% (*n*=10)	65.2% (229)
Insulin resistance	0.25	16	351	56.3% (*n*=9)	41.9% (147)
Hyperandrogenism [FAI]	0.74	16	351	43.8% (*n*=7)	39.6% (139)
Hypercholesterolemia	0.79	16	351	18.8% (*n*=3)	16.2% (57)
High LDL	0.12	16	351	0 (*n*=0)	7.4% (26)
Hypertriglyceridemia	0.88	16	351	6.3% (*n*=1)	5.4% (19)
Hyperprolactinemia	0.36	16	351	18.8% (3)	10.8% (38)
Hypovitaminosis D	0.69	16	351	81.3% (13)	77.2% (271)
TSH >2.5 and negative ATA (*n*=98, N=367)					
Variable	*p*	*n*	N-*n*	%*n*	% (N-*n*)
Pregnancy	0.81	98	269	8.2% (*n*=8)	7.4% (20)
Polycystic ovarian morphology	0.77	98	269	66.3% (*n*=65)	64.7 % (174)
Insulin resistance	0.01	98	269	54.1% (*n*=53)	38.3% (103)
Hyperandrogenism [FAI]	0.22	98	269	44.9% (*n*=44)	37.9% (102)
Hypercholesterolemia	0.06	98	269	22.4% (*n*=22)	14.1% (38)
High LDL	0.35	98	269	9.2% (*n*=9)	4.8% (13)
Hypertriglyceridemia	0.4	98	269	7.1% (*n*=7)	4.8% (13)
Hyperprolactinemia	0.07	98	269	16.3 % (16)	9.3% (25)
Hypovitaminosis D	0.13	98	269	82.7% (81)	75.5% (203)
TSH >2.5 regardless of ATA status (*n*=114, N=367)					
Variable	*p*	*n*	N-*n*	%*n*	% (N-*n*)
Pregnancy	0.94	114	253	7.9% (*n*=9)	7.5% (19)
Polycystic ovarian morphology	0.77	114	253	66.7% (*n*=76)	64.4% (163)
Insulin resistance	0	114	253	54.4% (*n*=62)	37.2% (94)
Hyperandrogenism [FAI]	0.26	114	253	44.7% (*n*=51)	37.6% (95)
Hypercholesterolemia	0.06	114	253	21.9% (*n*=25)	13.8 % (35)
High LDL	0.73	114	253	7.9% (*n*=9)	6.7% (17)
Hypertriglyceridemia	0.4	114	253	7% (*n*=8)	4.7% (12)
Hyperprolactinemia	0.03	114	253	14% (*n*=16)	8.7% (22)

Hypovitaminosis D	0.09	114	253	84.2% (*n*=96)	74.7% (188)
TSH ≤2.5 and negative ATA (*n*=225, N=367)					
Variable	*p*	*n*	N-*n*	%*n*	% (N-*n*)
Pregnancy	0.73	225	142	8% (*n*=18)	7% (10)
Polycystic ovarian morphology	0.73	225	142	64.4% (*n*=145)	66.2% (94)
Insulin resistance	0.04	225	142	38.2% (*n*=86)	49.3% (70)
Hyperandrogenism [FAI]	0.22	225	142	37.3% (*n*=84)	43.7% (62)
Hypercholesterolemia	0.03	225	142	12.9% (*n*=29)	21.8% (31)
High LDL	0.7	225	142	7.6% (*n*=15)	7.7% (11)
Hypertriglyceridemia	0.3	225	142	4.4% (*n*=10)	7% (10)
Hyperprolactinemia	0.04	225	142	8.4% (19)	15.5% (22)
Hypovitaminosis D	0.07	225	142	74.2% (167)	82.4% (117)
TSH ≤2.5 and positive ATA (*n*=28, N=367)					
Variable	*p*	*n*	N-*n*	%*n*	% (N-*n*)
Pregnancy	0.35	28	339	3.6% (*n*=1)	8% (27)
Polycystic ovarian morphology	0.75	28	339	67.6% (*n*=19)	64.9% (220)
Insulin resistance	0.11	28	339	28.6 % (*n*=8)	43.7% (148)
Hyperandrogenism [FAI]	0.95	28	339	39.3% (*n*=11)	39.8% (135)
Hypercholesterolemia	0.46	28	339	21.4% (*n*=6)	15.9 % (54)
High LDL	0.99	28	339	7.1% (*n*=2)	7.1% (24)
Hypertriglyceridemia	0.69	28	339	7.1% (*n*=2)	5.3% (18)
Hyperprolactinemia	0.93	28	339	10.7% (*n*=3)	11.2% (38)
Hypovitaminosis D	0.52	28	339	82.1% (*n*=23)	77% (261)
ATA regardless of the TSH result (*n*=44, N=367)					
Variable	*p*	*n*	N-*n*	%*n*	% (N-*n*)
Pregnancy	0.38	44	323	4.5% (*n*=2)	8% (26)
Polycystic ovarian morphology	0.9	44	323	66% (*n*=29)	65% (210)
Insulin resistance	0.57	44	323	38.6% (*n*=17)	43% (139)
Hyperandrogenism [FAI]	0.87	44	323	40.9% (*n*=18)	40% (128)
Hypercholesterolemia	0.44	44	323	20.5% (*n*=9)	15.7% (51)
High LDL	0.46	44	323	4.5% (*n*=2)	7.4% (24)
Hypertriglyceridemia	0.68	44	323	6.8% (*n*=3)	5.3% (17)
Hyperprolactinemia	0.59	44	323	13.6% (6)	10.8% (35)
Hypovitaminosis D	0.44	44	323	81.8% (36)	76.8% (248)
